# Isolated agenesis of the corpus callosum and normal general intelligence development during postnatal life: a case report and review of the literature

**DOI:** 10.1186/s13256-020-2359-2

**Published:** 2020-02-12

**Authors:** Parménides Guadarrama-Ortiz, José Alberto Choreño-Parra, Tania de la Rosa-Arredondo

**Affiliations:** Department of Neurosurgery, Centro Especializado en Neurocirugía y Neurociencias México (CENNM), Tlaxcala & Manzanillo, Roma Sur, 06760 Mexico City, Mexico

**Keywords:** Agenesis of the corpus callosum, Cerebral malformations, Intellectual disability, Neurocognitive development, Neuropsychological testing, Ventriculomegaly

## Abstract

**Background:**

Agenesis of the corpus callosum can occur isolated or as part of a complex congenital syndrome. Patients with isolated agenesis of the corpus callosum may present with severe intellectual disability, although a proportion of affected individuals develop normal intelligence. However, even in patients with no apparent deficits, subtle neuropsychological alterations may occur as the cognitive demand increases with age. Hence, patients with this deffect require a strict follow-up during their postnatal life. Thus, physicians require a better knowledge of the cognitive features of agenesis of the corpus callosum to improve their approach to this cerebral malformation. Here, we report an illustrative case of a school-age child with isolated agenesis of the corpus callosum and normal intelligence. We also provide a literature review about the postnatal screening of neurocognitive deficits in patients with agenesis of the corpus callosum.

**Case presentation:**

An 8-year-old Hispanic boy with total agenesis of the corpus callosum attended for medical follow-up. The defect was identified during the neonatal period by cranial ultrasonography and brain computed tomography scan. However, he did not present any craniofacial or non-cerebral malformation suggestive of a congenital syndrome. Furthermore, he showed no neuropsychiatric disorder or intellectual disability during his early childhood. At the age of 4, he was subjected to a control brain magnetic resonance imaging that showed total agenesis of the corpus callosum and colpocephaly. At his arrival, a neurological examination was normal with no signs of intracranial hypertension. His intelligence quotient was unaltered and he scored normal in the Mini-Mental State Examination test. The literature reviewed here suggested that patients with agenesis of the corpus callosum require a strict neurocognitive follow-up during postnatal life, as they may present neuropsychological deficits during adolescence, when development of the corpus callosum is completed and there is maximum reliance on this structure. Thus, our patient was scheduled for future annual neurocognitive testing.

**Conclusions:**

Isolated agenesis of the corpus callosum is not innocuous, and patients with this defect require a strict neurocognitive follow-up. We provide an informative reference tool useful for the postnatal neuropsychological screening of patients with isolated agenesis of the corpus callosum.

## Background

Agenesis of the corpus callosum (AgCC) is a rare brain malformation that can occur isolated or associated with other anatomical defects as part of a complex congenital syndrome [1]. The defect is “complete” when total absence of the corpus callosum (CC) occurs or “partial” when only certain regions of the structure are formed. Despite the gross anatomical consequences of this defect, the spectrum of neurological manifestations observed in individuals with AgCC varies from severe intellectual disability to normal intelligence [2–4]. However, even in individuals with isolated AgCC and no evident neurological deficit, subtle neuropsychological alterations may occur as the cognitive demand increases with age [5, 6]. Therefore, a long-term follow-up by adequate neuropsychological screening is required for all cases of AgCC and “normal” intellectual quotient (IQ). Unfortunately, because of the rarity of this malformation, knowledge of the neurological and cognitive features of AgCC is limited among physicians, which may lead to a late identification of neurocognitive alterations and to a delayed establishment of rehabilitative strategies aimed to enhance compensatory functions in affected individuals.

Here, we describe the case of a school-age child with isolated total AgCC and apparent grossly intact general intelligence. In addition, we summarize the relevant literature about this cerebral defect, focusing on the diagnostic approach and neuropsychological screening during postnatal life. Our study provides an informative quick reference tool useful for the longitudinal postnatal cognitive evaluation and follow-up of patients with isolated AgCC.

## Case presentation

**Fig. 1:**
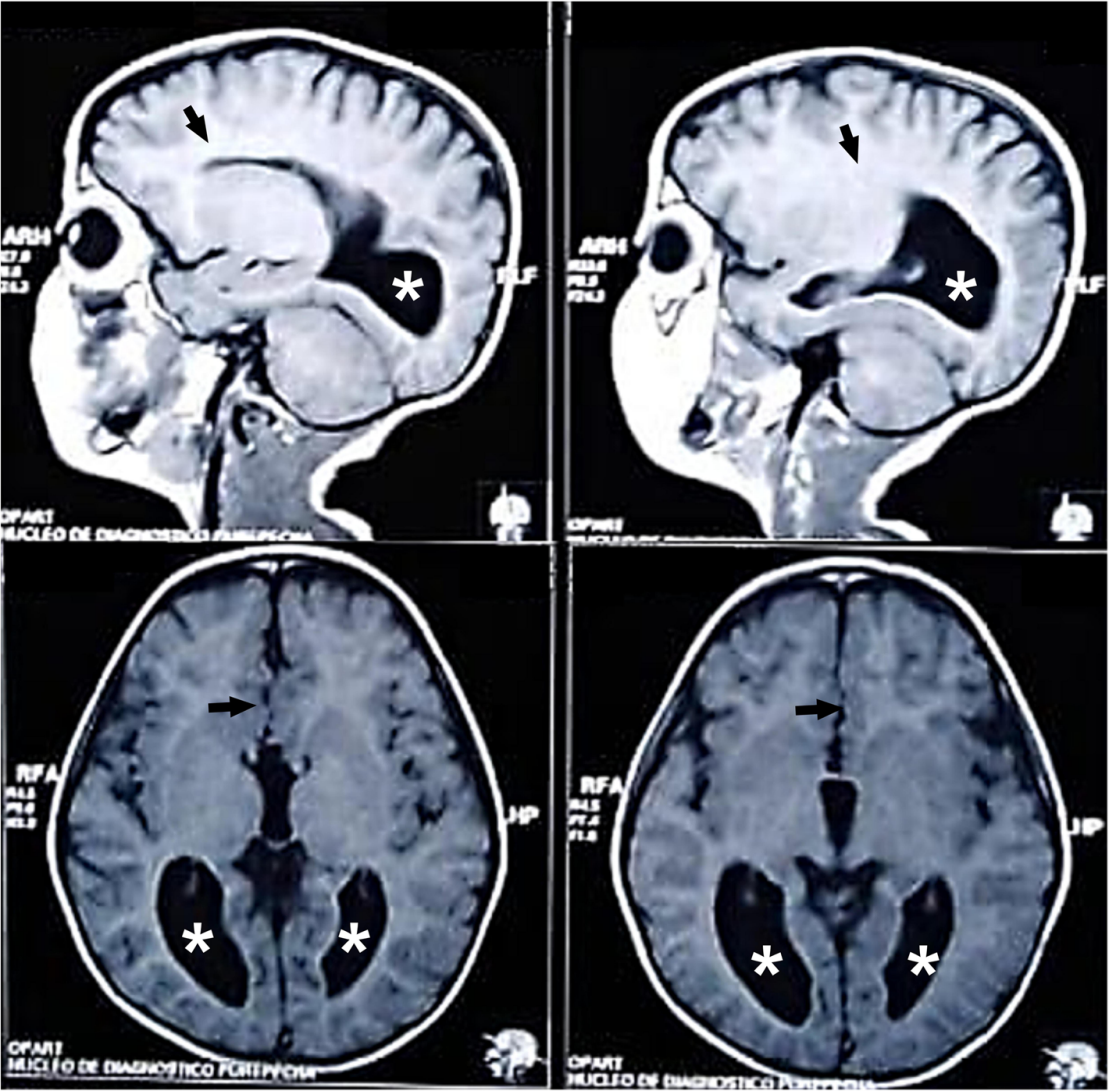
Brain magnetic resonance imaging images of the clinical case. Sagittal (*upper panels*) and transversal (*lower panels*) T1-weighted brain magnetic resonance imaging images showing total agenesis of the corpus callosum (*black arrows*) and enlargement of the occipital horns of the lateral ventricles (*white stars*).

An 8-year-old Hispanic boy attended our center to receive a follow-up medical examination because his mother had stated that he was diagnosed as having AgCC during the neonatal period. He was the product of her first gestation, delivered by caesarean section at 36 weeks due to hydrocephalus, with an Appearance, Pulse, Grimace, Activity, and Respiration (APGAR) score of 8/10 at 1 and 5 minutes after birth, and no perinatal complications. His mother denied history of congenital infection and teratogenic exposures during pregnancy. The child was subjected to a postnatal cranial ultrasonography (USG) that revealed total AgCC; thus, a computed tomography (CT) scan of his brain was performed which confirmed the diagnosis. Further genetic screening and metabolic screening were not performed, but the child did not present any unusual craniofacial, digital, or neurocognitive feature suggestive of a congenital syndrome or any other non-cerebral structural abnormality. Also, no evidence of visual or hearing impairment was observed. His pattern of postnatal growth and neurocognitive development was normal. Furthermore, he did not show any neurological sign, behavioral or psychiatric disorder, or intellectual disability during his early childhood and school age. His school performance was not affected, and he was able to practice taekwondo. The rest of his past medical history was relevant only for allergic contact dermatitis. At the age of 4, he was subjected to a control magnetic resonance imaging (MRI) of his brain that showed total AgCC, as well as enlargement of the lateral ventricles with dilated occipital horns (colpocephaly; Fig. 1). However, he did not present any clinical data of intracranial hypertension.

During the medical examination he was alert, aware, oriented, and asymptomatic. His language was fluent, and his posture was normal with no abnormal movements. He was able to follow simple commands and to name objects. He did not present evidence of any apraxia or agnosia. A physical examination revealed normal gait, and no alterations in cranial nerve reflexes, muscle strength, deep tendon reflexes, and plantar reflexes. An examination of the cerebellum did not show any abnormality, and no clinical features of intracranial hypertension and meningism were observed. The child scored normal in the Mini-Mental State Examination (MMSE) test, and his IQ was within the normal range. His weight and height were in the 50th percentile according to his age and gender. Blood tests, including thyroid, liver, and renal function, as well as electrolyte and vitamin panel were normal. Finally, non-pharmacological management was prescribed, and he was scheduled for annual follow-up medical appointments for future neurocognitive screenings at our center. The legal guardian of the patient provided written informed consent for the publication of the case.

## Discussion and conclusions

The CC is the largest cerebral white matter commissure consisting of approximately 200 million axons connecting different parts of the right and left hemispheres [7]. This structure is divided in five anatomical regions: genu, rostrum, body, isthmus, and splenium. The CC contains homotopic and heterotopic interhemispheric nerve tracts establishing both excitatory and inhibitory connections. This allows bilateral communication between primary sensory-motor areas and integration of cortical nuclei located on the contralateral or ipsilateral hemisphere [8]. Transcallosal fibers are organized in separate nerve tracts along the subregions of the CC according to the functional areas toward which they are projected: prefrontal; premotor and supplementary motor; primary motor; primary sensory; parietal lobe; temporal lobe; and occipital lobe [9].

The connecting role of the CC is of great relevance for the integration of input information necessary for adequate neurocognitive functioning [10]. Thus, damage to the CC during postnatal life or its congenital absence causes important neuropsychological deficits according to the anatomical subregions compromised (Table 1). A spectrum of structural malformations can affect the CC, including complete or partial AgCC, hypoplasia, and dysgenesis [16]. AgCC is one of the most common congenital brain defects and occurs in 1.4 per 10,000 live births [17]. This defect results from the disruption of CC development which begins at around the tenth week of gestation with the migration of glial cells to the interhemispheric fissure, where they form a primitive glial sling [18, 19]. This structure guides callosal fibers which cross to the opposite hemisphere along two separate loci: one containing axons from the anterior hemispheric neocortex and a second one formed by fibers from the posterior neocortex. This process is regulated by several genes involved in the target recognition and migration of axons to the contralateral hemisphere [20]. The formation of the CC culminates with the fusion of the anterior and posterior loci at the 20th week of gestation [19].

**Table 1:** Neurocognitive features of disrupted interhemispheric connectivity in patients with corpus callosotomy and isolated agenesis of the corpus callosum

Region of the CC	Nerve-fiber tracts	Clinical features in patients with postnatal non-congenital CC lesions (References [11–13])	Neurocognitive testing^a^ (Reference [14])	Cognitive deficiencies in patients with isolated AgCC^b^ (Reference [5])	Neuropsychological testing^c^ (References [5, 15])
Genu and rostrum	Prefrontal	Non-dominant alien hand syndrome	Tactile Object Recognition test Hand Pose Imitation test Inter-Manual Localization of Pressure Points test Imitation of one-handed transitive and intransitive gestures drawn Tachistoscopic bilateral visual field matching task	Incoordination of both hand movements Slow sensory and motor reaction times Difficult spontaneous memory retrieval Difficult learning of novel and unfamiliar verbal and visual information Impaired reasoning, concept formation, and novel complex problem solving Poor comprehension of sarcasm Limited interpretation of second-order meanings Deficient cognitive inhibition and flexibility Deficient formulation of strategies Defective application of imagination and creativity Poor interpretation and expression of emotions according to the social context Deficient social communication	Wide Range Achievement Test 3 (WRAT-3) for academic skills in the areas of spelling, reading, and arithmetic 6-block and the 10-block versions of the Tactile Performance Test (TPT) Bimanual Coordination Test (BCT) Tactual Performance Test Finger Localization Test Raven’s Color Progressive Matrices for complex problem solving based on primarily visual/spatial stimuli Letter and Number Series Tests from the Primary Mental Abilities Test for complex problem solving and inductive reasoning ability Minnesota Multiphasic Personality Inventory-2 (MMPI-2) 12-item and 40-item free-answer version of the Proverbs Test for abstract verbal comprehension and reasoning The Thematic Apperception Test (TAT) for recognition of social situations The Rorschach Inkblot Test for conventional visual recognition and interpretation of ambiguous information The Child Behavior Checklist (CBCL) for behavioral problems
Body and isthmus	Premotor	Left unilateral motor apraxia Right unilateral constructional apraxia Impaired rapid alternating movement of both hands Left hand agraphia Left tactile anomia Left auditory anomia Right olfactory anomia			
	Supplementary motor				
	Primary motor				
	Primary sensory				
	Parietal lobe				
	Temporal lobe				
Splenium	Occipital lobe	Left hemialexia Left visual anomia Pure alexia	Reading words aloud Word reading Oral naming of visual presentation Written naming of visual presentation		

^a^Tests used for the evaluation of disconnection interhemispheric syndrome in patients with postnatal lesions of the corpus callosum that may be helpful to reveal neurological deficits in patients with isolated agenesis of the corpus callosum. ^b^The pattern of cognitive deficiencies may vary between patients with isolated agenesis of the corpus callosum as a result of other clinical factors. ^c^Tests used for the screening of neuropsychological deficits in patients with isolated agenesis of the corpus callosum and apparent normal intelligence. Always include the Mini-Mental State Examination, the Wechsler Intelligence Scale for Children, and the Wechsler Adult Intelligence Scale, as well as other validated tests used according to the age and language of the patient. *AgCC* agenesis of the corpus callosum, *CC* corpus callosum

AgCC can occur as an isolated condition or can be associated with other brain and extracranial malformations as part of a wide range of congenital syndromes related to known teratogenic infectious, toxic, or metabolic exposures, as well as genetic disorders. In fact, 10% of individuals with AgCC have chromosomal anomalies and 20–35% have specific monogenic or polygenic disorders [21]. Fetal alcohol syndrome (FAS) is the most important non-genetic congenital cause of AgCC, with an incidence of approximately 7% in FAS cases [22]. Yet, in approximately 70% of the cases of AgCC, especially in those of complete isolated agenesis, the causative condition remains unknown [21, 23, 24]. The absence of CC disrupts interhemispheric communication and confines the functional processing network of complex cognitive functions [6]. However, there is not a single neuropsychological phenotype of individuals with AgCC. In fact, the spectrum of clinical features of this defect is heterogeneous and depends on several factors, such as the extension of the CC damage (complete or partial), the presence and severity of associated defects, clinical comorbidities, as well as on the magnitude of residual interhemispheric transfer through other commissures (anterior, posterior, and hippocampal) [5]. Probst and heterotopic bundles also develop in patients with AgCC and may provide a certain degree of compensatory connectivity [25, 26].

In general, patients with AgCC can be categorized into three groups according to their clinical and neurocognitive characteristics. The first includes individuals with “syndromic” AgCC who commonly present severe neurocognitive and intelligence disability which obscures the deficiencies directly caused by the absence of the CC. These patients can also present evident associated brain malformations, non-cerebral structural defects, altered patterns of growth and development, progressive neurological symptoms, sensory impairment, and systemic symptoms [16]. A second group of patients with neurodevelopmental diseases in which AgCC may play a role has been suggested [27]. The third group, illustrated by the case presented here, includes patients with isolated complete or partial AgCC who typically remain neurologically asymptomatic and have apparent normal intelligence. However, recent studies have shown that a deep neuropsychological screening often reveals mild behavioral and cognitive deficits in these individuals [5].

**Fig. 2:**
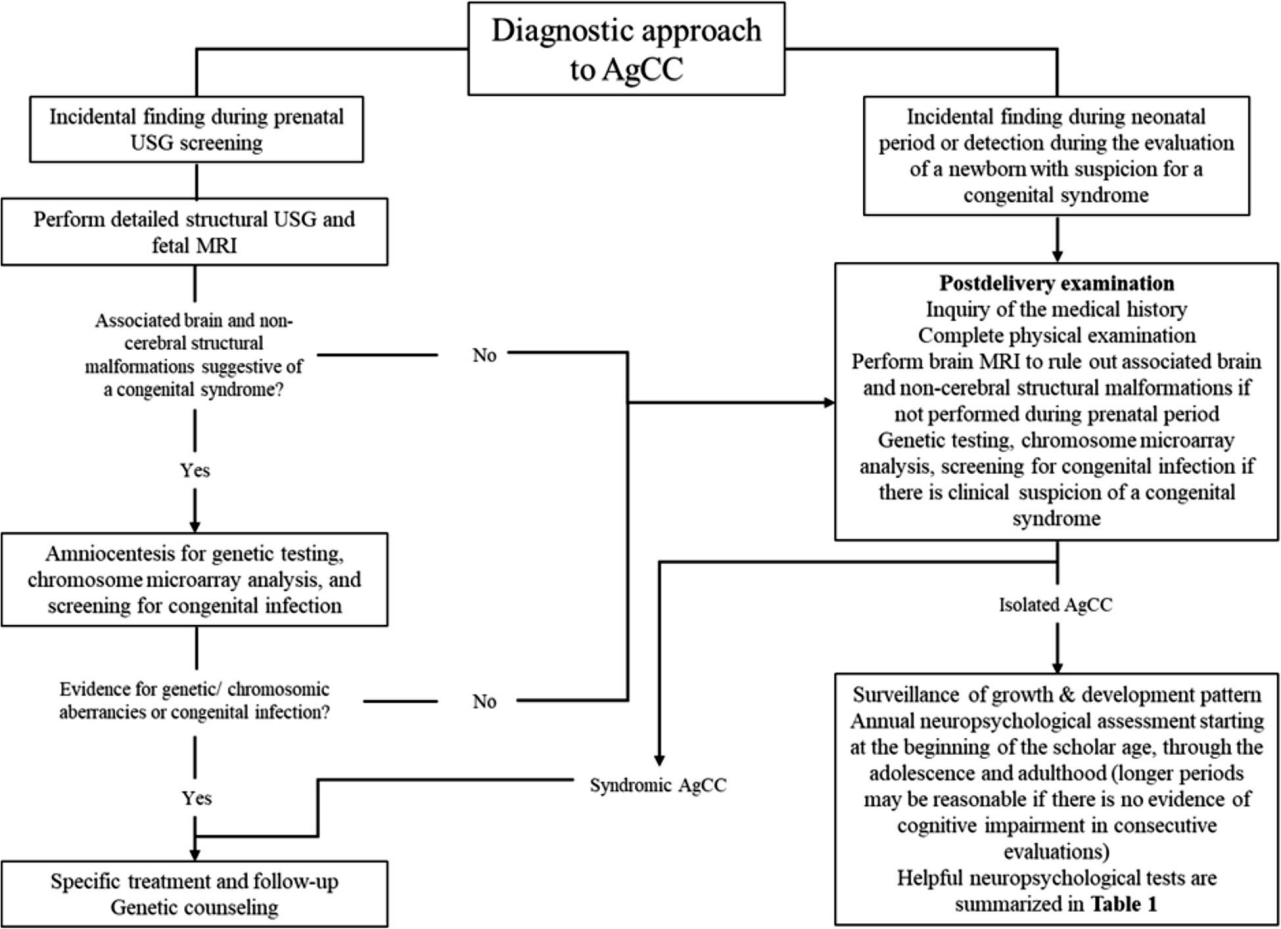
Prenatal and postnatal diagnostic and follow-up approach to agenesis of the corpus callosum. *AgCC* agenesis of the corpus callosum, *MRI* magnetic resonance imaging, *USG* ultrasonography

The diagnosis of AgCC can be performed during the prenatal or postnatal period. Prenatal diagnosis is commonly performed using USG between 18 and 22 weeks of gestation, which might directly reveal the complete or partial defect, as well as other indirect features suggestive of AgCC [28]. This approach must be complemented with a detailed structural USG and a prenatal brain MRI to confirm the presence of AgCC and other possible associated defects [28]. A brain MRI could be repeated after birth to improve the screening of accompanying malformations. Amniocentesis for genetic testing and chromosome microarray analysis, as well as screening for congenital infections are recommended, especially in those with multiple defects detected by imaging [1]. Mothers who have a prenatal diagnosis of AgCC must be managed by a multidisciplinary team. During the early postnatal period, all the previous studies must be complemented with: a full inquiry of the medical history; a complete physical examination; imaging of gastrointestinal, respiratory, urinary, and cardiovascular systems; as well as additional studies, such as a metabolic panel, somatosensory evoked potentials test, among others, especially in cases with clinical features suggestive of syndromic AgCC (Fig. 2) [1, 28].

Even when some individuals with syndromic AgCC present evident neurological symptoms and cognitive decline since early life, all patients must receive a longitudinal neuropsychological screening and follow-up until adulthood. Patients with isolated AgCC and apparent normal intelligence constitute the population that would benefit the most from an opportune detection of subtle neurocognitive anomalies, as they can receive timely rehabilitative strategies to compensate such deficits, which would improve their independency. These patients often present neuropsychological defects at the end of childhood and beginning of adolescence, when myelination and development of the CC is completed and the reliance on this structure becomes maximum [18, 29]. Before such a moment, the presence of other cerebral commissures provides a certain degree of compensatory connectivity [25, 26]. However, individuals with AgCC and apparent normal intelligence become more susceptible to increases in cognitive demand over time. Their neurocognitive phenotype results from a lack of interhemispheric transfer of complex or unfamiliar information, but normal connectivity for the transmission of simple sensory-motor inputs. This disrupted connectivity also leads to a slow processing speed of sensory-motor information, which is exacerbated as the complexity of different tasks requires a higher cognitive demand [5]. The explanation for this limitation in the processing of complex information and relatively normal performance in simple or familiar cognitive functions is that such complex tasks often require the simultaneous recruitment of integration centers located in both hemispheres [30]. Interestingly, individuals with AgCC also have a marked limitation to integrate or interpret social and emotional information according to the context [5].

Collectively, these deficits cause a range of secondary neurocognitive manifestations that have recently been proposed to constitute the core syndrome of AgCC [5]. Some of these deficits may improve with training and practice, which highlights the importance of neuropsychological screening to timely initiate neurocognitive strategies aimed to enhance compensatory mechanisms. In Table 1 we provide a summary of the cognitive findings that might be observed in apparently normal patients with AgCC, as well as the neuropsychological tests used in the past for the investigation of such neuropsychological deficits. The battery of neurocognitive tests must always include general neuropsychological tools such as the MMSE, as well as other validated tests used according to the age and language of the patient. The Wechsler Intelligence Scale for Children (WISC) and the Wechsler Adult Intelligence Scale (WAIS) must also be performed to evaluate the IQ of the patients [31, 32]. Furthermore, due of the lack of evidence-based recommendations for the follow-up of individuals with isolated AgCC, here we propose that this kind of patients should be evaluated at least yearly, starting at the beginning of school age, to timely detect any deficit. It is important to mention that, although children with isolated AgCC can show normal performance during their initial evaluations, these early examinations may provide valuable reference information for future testing.

In conclusion, AgCC is not an innocuous brain malformation even if it occurs isolated, and patients with this defect require a strict neurocognitive follow-up despite normal cognitive function during childhood, as illustrated in the current report. Although the relevance of the case presented here does not rely on any unusual clinical presentation of AgCC, the rarity of the defect, as well as the late age of our patient’s attendance, makes this report a clear demonstration of the compensatory capacity of the brain in the absence of CC that allowed our patient to experience a relatively normal childhood. Finally, we believe that the current review would provide physicians with a reference tool useful for the diagnostic approach and neuropsychological follow-up of patients with isolated AgCC, which ultimately can contribute to improve their independency and integration to social life.

## Data Availability

The clinical data from the case presented here are available from the corresponding author on reasonable request.

## Abbreviations

AgCC: Agenesis of the corpus callosum
APGAR: Appearance, Pulse, Grimace, Activity, and Respiration
CC: Corpus callosum
CT: Computed tomography
FAS: Fetal alcohol syndrome
IQ: Intellectual quotient
MMSE: Mini-Mental State Examination
MRI: Magnetic resonance imaging
USG: Ultrasonography
WAIS: Wechsler Adult Intelligence Scale
WISC: Wechsler Intelligence Scale for Children
